# Direct transfer of HRPII-magnetic bead complexes to malaria rapid diagnostic tests significantly improves test sensitivity

**DOI:** 10.1186/s12936-016-1448-6

**Published:** 2016-08-05

**Authors:** Keersten M. Ricks, Nicholas M. Adams, Thomas F. Scherr, Frederick R. Haselton, David W. Wright

**Affiliations:** 1 Department of Chemistry, Vanderbilt University, Nashville, TN 37235 USA; 2 Department of Biomedical Engineering, Vanderbilt University, Nashville, TN 37235 USA

**Keywords:** Malaria, Diagnostics, Extraction, Magnetic beads, Biomarker concentration

## Abstract

**Background:**

The characteristic ease of use, rapid time to result, and low cost of malaria rapid diagnostic tests (RDTs) promote their widespread use at the point-of-care for malaria detection and surveillance. However, in many settings, the success of malaria elimination campaigns depends on point-of-care diagnostics with greater sensitivity than currently available RDTs. To address this need, a sample preparation method was developed to deliver more biomarkers onto a malaria RDT by concentrating the biomarker from blood sample volumes that are too large to be directly applied to a lateral flow strip.

**Methods:**

In this design, Ni–NTA-functionalized magnetic beads captured the *Plasmodium falciparum* biomarker HRPII from a *P. falciparum* D6 culture spiked blood sample. This transfer of magnetic beads to the RDT was facilitated by an inexpensive 3D-printed apparatus that aligned the sample tube with the sample deposition pad and a magnet beneath the RDT. Biomarkers were released from the bead surface onto the lateral flow strip using imidazole-spiked running buffer. Kinetics of HRPII binding to the Ni–NTA beads as a function of blood sample volume were explored prior to determining the effect of the proposed method on the limit of detection of Paracheck RDTs.

**Results:**

More than 80 % of HRPII biomarkers were extracted from blood sample volumes ranging from 25 to 250 µL. The time required to reach 80 % binding ranged from 5 to 60 min, depending on sample volume. Using 250 μL of blood and a 30-min biomarker binding time, the limit of detection of the Paracheck Pf RDT brand was improved by 21-fold, resulting in a limit of detection below 1 parasite/μL.

**Conclusions:**

This approach has the sensitivity and simplicity required to assist in malaria elimination campaigns in settings with limited access to clinical and laboratory resources.

**Electronic supplementary material:**

The online version of this article (doi:10.1186/s12936-016-1448-6) contains supplementary material, which is available to authorized users.

## Background

Malaria is a treatable infectious disease caused by a parasite of the genus *Plasmodium.* In 2014, 97 countries and territories were documented to have ongoing malaria transmission, and 3.3 million people are estimated to be at risk of infection. As the global health community presses toward malaria elimination, the need for more sensitive point-of-care diagnostic tests becomes essential. Malaria rapid diagnostic test (RDT) demand has grown significantly in recent years. These lateral flow-based tests, which operate much like a commercial pregnancy test, detect protein biomarkers of malarial infection in a blood sample. The estimated need for malaria RDTs, in Africa alone, is 1.6 billion between the years of 2013–2016 [1]. Compared to other diagnostic strategies (i.e., microscopy, traditional immunoassays and PCR), RDTs are more easily deployed for point-of-care testing because they are cost effective (<$1.50 per test), easy to use, tolerant of a wide range of environmental conditions, and offer a rapid time-to-result [2]. While they have been useful in malaria control campaigns, the majority of commercially available RDTs lack the sensitivity required to detect asymptomatic or submicroscopic infections (<200 parasites/µL of blood), which is critical for moving from malaria control to elimination campaigns [3–5]. Although it is unclear what degree of improvement in sensitivity is required for RDTs to be useful in elimination campaigns, there seems to be consensus from many groups that the 0.1–2 parasites/µL blood detection limit of polymerase chain reaction (PCR) is sufficient [6–10]. Furthermore, diagnostic tools with detection limits of 1 parasite/µL blood have been cited as effective for active case detection and population surveillance [11]. Based on these values, it was anticipated that if the sensitivity RDTs can be improved to detect ~1 parasite/µL of blood, the current RDT-based approach for case management at the point-of-care has the potential to be effective for malaria elimination.

A number of strategies have been reported for enhancing the sensitivity of commercial RDTs, many of which focus primarily on amplifying the signal at the test line [12–14]. Others have explored RDT enhancement approaches based on the hypothesis that if an increased number of biomarkers can be delivered to the test from larger sample volumes without altering the function of the test, an enhancement in diagnostic capability would be observed [15–18]. The development of a magnetic bead-based sample preparation method was recently reported to purify and concentrate malaria biomarkers from whole blood [19, 20]. It was demonstrated that the predominate biomarker of *Plasmodium falciparum* infection, histidine-rich protein II (HRPII) could be extracted from a 100-µL whole blood sample with high efficiency and specificity using Ni–NTA functionalized magnetic particles, purified through a series of preloaded processing solutions in a length of tubing, and concentrated into a 10-µL volume of elution buffer for downstream detection. Following the processing steps, the elution buffer was pipetted onto an RDT [19]. While the efficacy of this method for purification of *pf*HRPII from whole blood was initially evaluated by traditional protein detection methods (e.g., gel chromatography and ELISA), a significant increase in commercial malaria RDT signal was observed when the concentrated product was pipetted onto the test. This approach resulted in a fourfold to 13-fold enhancement in the limit of detection compared to RDTs performed using 5-µL blood samples as suggested by the manufacturer [20]. This proof of concept successfully demonstrated enhancement of unmodified commercial malaria RDTs, but the multi-step design of the protein purification method and the need for a pipette-based transfer step was at odds with the characteristic simplicity and field-deployable nature of RDTs.

**Fig. 1:**
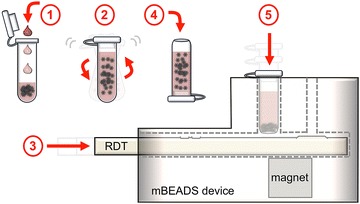
Schematic of the biomarker extraction and delivery system. (*1*) Add blood to sample tube containing magnetic beads and lysis reagents. (*2*) Mix the sample using a handheld, battery-powered vortex for 5–30 min to bind biomarkers. (*3*) Insert an RDT into the 3D printed transfer device. (*4*) Transfer the sample to a cut-bottom dispensing tube. (*5*) Insert the dispensing tube into the transfer device and wait 30 s for the beads to collect near the bottom before depressing fully to blot the beads onto the test. The RDT is then developed using imidazole-spiked development buffer to release the biomarkers from the beads

To maintain this ease-of-use which defines malaria RDTs but to still achieve enhancement in diagnostic ability, a new design was developed to: (i) directly transfer the HRPII-bead complex to the RDT in a single processing step; and, (ii) release the HRPII directly onto the RDT (Fig. 1). The major advantages of this approach are that it does not replace the well-accepted and familiar RDT, but improves it with the addition of a simple bead-based concentration approach and additional user steps. Key variables of this new approach were explored to understand the effects of sample volume, imidazole concentration, and bead mixing time on RDT performance and to determine if the limit of detection required for elimination campaigns could be achieved.

## Methods

### Materials

Ni–NTA Magnetic Agarose Beads (cat # 36111) were purchased from Qiagen Inc. Pooled human whole blood collected in citrate phosphate dextrose (CPD) anticoagulant (cat # HMWBCPD) was purchased from Bioreclamation LLC. D6 *P. falciparum* stock culture was prepared in the Wright laboratory (18,450 parasites/µL stock concentration). Paracheck Pf RDTs (cat # 30301025) were acquired from Orchid Biomedical Systems. Flat-cap 500 µL PCR tubes (cat # AB-0350) were purchased from Fisher Scientific. mBEADS dispensing tubes were prepared by cutting the end off of 500 µL PCR tubes at ~1.5 mm from the bottom of the tube (the end opposite of the cap), leaving an ~2.5 mm diameter hole in the bottom at the tube. The mBEADS transfer device was printed on a ProJet HD 3000 Plus 3D printer (an image of the device is provided in Additional file 1: Figure S1). The Qiagen ESEQuant Lateral Flow Reader and software was acquired from Qiagen Lake Constance. All other materials and reagents were purchased from Fisher Scientific.

### Image analysis of blood clearance from the lateral flow strip

In order to examine the inherent purification capabilities of the lateral flow strip, blood volumes of 0, 5, 10, 15, 20, 25, 50, and 100 µL, each containing 200 parasites/µL, were run on Paracheck RDTs according to the manufacturer’s recommended procedure. A Nikon D800 36.3 MP D-SLR camera (Nikon Inc., Melville, NY, USA) with a AF-S Nikkor 24–85 mm f/3.5-4.5G ED VR lens (Nikon Inc., Melville, NY, USA) was positioned above the RDTs, and was sufficiently elevated to avoid shadowing the RDTs below. The camera was used in time-lapse mode to collect images every 4 s for 30 min. The resulting images were read into a custom image analysis program developed in MATLAB v8.5 (MathWorks, Natick, MA, USA). For each frame, two regions of interest were manually selected on the RDTs. Average pixel intensity inside a preselected region upstream of both the control and test lines on the membrane was measured as the test area, and average pixel intensity from a second region on the plastic cartridge of the RDT was use for white-balance correction. White balance normalization of the test area was calculated as $\tilde{I}_{T} = {{\left( {\bar{I}_{W} - \bar{I}_{T} } \right)} \mathord{\left/ {\vphantom {{\left( {\bar{I}_{W} - \bar{I}_{T} } \right)} {\bar{I}_{W} }}} \right. \kern-0pt} {\bar{I}_{W} }}$, where $\tilde{I}_{T}$ is the normalized pixel intensity of the test area, *Ī*_*W*_ is the average pixel intensity of the white-balance area, and *Ī*_*T*_ is the average pixel intensity of the test area.

### Effect of sample volume on binding kinetics of HRPII to Ni–NTA magnetic particles

Samples were prepared by spiking whole blood with D6 *P. falciparum* parasite culture to a final concentration of 200 parasites/µL, in order to closely mimic a patient sample. The blood was lysed at a 1:1 ratio with lysis buffer, and 50, 100, 200, or 500 µL volumes of lysed blood plus 20 µL of Ni–NTA magnetic particles were added to 1.5 mL Eppendorf tubes (n = 3 for each time point). The manufacturer-estimated binding capacity of 20 µL of bead suspension (40 µg) was estimated to be in great excess of the amount of HRPII present. The tubes were placed on a bench top vortexer fitted with a tube holder attachment and vortexed for 5, 10, 15, 30, or 60 min at medium speed. For each time point, three tubes from each sample volume were spun down on a minicentrifuge for 10 s to collect the sample and beads at the bottom of the tube. The tubes were then placed on a magnetic tube rack to pull the magnetic particles to the side of the tube while the blood sample supernatant was collected. The blood supernatant samples for each sample volume at each time point were analyzed by ELISA for HRPII content, using a previously published method [19].

Using these binding kinetics data, and with the assumption that mass transfer to the bead surface is rapid, the dissociation of bound biomarker is negligible, a constant volume is maintained, the system is well-mixed, and that the surface density of Ni–NTA is always in excess and its concentration remains in excess, a mole balance on free HRPII is solved to yield:

1 $$\frac{{N_{HRPII} }}{{N_{HRPII}^{0} }} = e^{{\frac{ - Kt}{V}}}$$

where *N*_*HRPII*_^0^ is the initial moles of biomarker, and K is a lumped constant proportional to the forward reaction rate constant, k, and the initial moles of Ni–NTA given by the product of the number of magnetic beads and the surface density of Ni–NTA. To determine the kinetic binding parameter, *K*, the fraction of HRPII remaining as a function of time and sample volume was fit using a non-linear least squares method in the Curve Fitting Toolbox of MATLAB v8.5 (MathWorks, Natick, MA, USA).

### Effect of sample volume on limit of detection of Paracheck RDT

Paracheck Pf RDTs were chosen for these studies because they have been identified by the WHO as a top performing test brand [21], they have been demonstrated to have relatively reliable quality standards and high sensitivity [19], and they can be obtained for research use in the USA. The limit of detection of the magnetic bead-enhanced Paracheck Pf RDTs was determined after concentrating biomarkers from blood sample volumes of 25, 50, 100, or 250 µL lysed blood spiked with increasing parasite concentrations corresponding to the linear range of the RDT’s signal response curve from that volume (0, 5, 10, 25, and 50 parasites/µL for the 50 µL sample volume series, 0, 1, 5, 10, and 25 parasites/µL for the 100 µL sample volume series, 0, 1, 5, 10, and 25 parasites/µL for the 200 µL sample volume series, and 0, 0.5, 1, 5, and 10 parasites/µL for the 500 µL sample volume series). Each sample concentration was run in triplicate. The samples were lysed at a 1:1 ratio of whole blood to lysis solution and incubated with 20 µL of Ni–NTA magnetic beads for 30 min on a benchtop vortexer. During mixing, the RDT was removed from the packaging and inserted into the transfer device. After mixing, the lysed blood and bead sample was transferred into the cut opening of a dispensing tube using a plastic transfer pipette. The dispensing tube was inverted cut-side-down and placed in the sample port of the transfer device. With the cap closed, the surface tension of the meniscus at the bottom of the dispensing tube was sufficient to retain the sample inside the tube. The magnetic beads were collected at the bottom of the tube by the magnet positioned below the RDT for 30 s, and then the dispensing tube was gently depressed ~2 mm to blot the beads on to the sample pad. The RDT was removed from the transfer device prior to adding to the buffer port 90 µL of the manufacturer-provided running buffer spiked with 500 mM imidazole. The RDT developed for 30 min. The lateral flow strip was then removed from the cassette and the peak area of the test line was measured using a Qiagen ESEQuant LFR. The average signal from the triplicate runs for each parasite concentration was plotted, and the linear regression was calculated and normalized by subtracting the average signal of the 0 parasite/µL blank. The lower limit of detection was calculated using the linear regression (*y* = *ax* + *b*) for each sample volume tested by solving for the parasite concentration (*x*) at the limit of blank (*y*). The *limit of blank* was calculated as the mean of the blank measurements plus three times the standard deviation of the 0 parasite/µL measurements. The limit of detection of the Paracheck Pf RDTs performed using 5 µL blood samples without biomarker enhancement was determined using the same method.

### Paracheck peak area analysis by Qiagen ESEQuant LFR

Using the LR Studio software supplied with the reader, a method was set up to read lateral flow strips from Paracheck RDTs. To do this, a Paracheck RDT was developed using a 2000 parasites/µL sample. After developing the test, the lateral flow strip was removed from the plastic housing, the wicking pad removed, and the strip was then placed in the LFR tray with the wicking end of the strip against the left edge of the tray. In the software, the channel and type was set to E1/D2 and reflective, respectively. The LFR was set to read from 0 to 60 mm, and the signal noise threshold was set to 1 mV. The software was also set to evaluate peak area at a fixed baseline, measuring left and right 1.5 mm. After entering these parameters, the green arrow was clicked to scan the strip. After the scan was complete, the vertical trace was placed over the center of the control line before double clicking on the ‘Pos [mm] box’. This parameter was saved as the ‘Control’. After adding a new parameter (Test → Add New Parameter), this process was repeated to set up the ‘Test’ line reading. Both the Control and Test parameters were then saved as the Paracheck method file. This method file was used to analyse and calculated the peak areas for each of the processed Paracheck RDTs.

## Results

### Effect of increased blood sample volume on RDT clearance

**Fig. 2:**
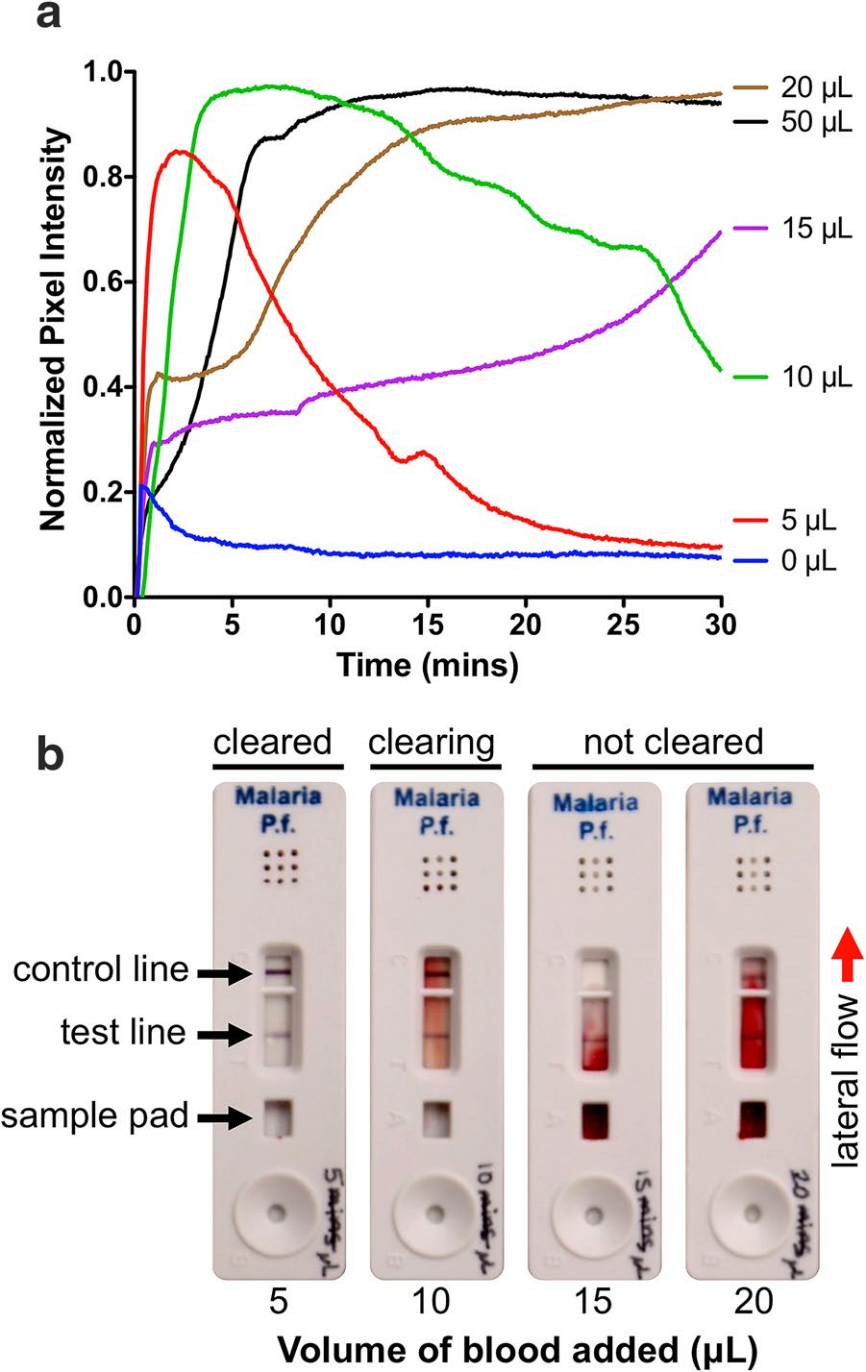
Image analysis of the clearance of blood from Paracheck Pf RDTs over a 30-min time course after adding 5, 10, 15, 20, or 50 µL of blood directly to the test. **a** Pixel intensity increases as blood flows onto the lateral flow strip and decreases as the blood is cleared from the strip by the running buffer. **b** Representative images of RDTs performed with 5, 10, 15, and 20 µL blood after 30 min. Proper RDT development is assessed by visualizing blood clearance from the sample pad and the area around the test and control lines. Time-lapse video of RDT clearance is available as an additional file online

In the case of biomarker-based malaria diagnostics, using larger patient blood sample volumes has the potential to improve the probability of detecting low abundance parasite biomarkers. Current RDTs rely on lancets to collect a small drop of blood (5–10 µL), however, with the increasing availability of simple blood collection techniques using ‘high-flow’ lancets, it has been demonstrated that blood volumes of 250 µL or more can be reliably collected from finger pricks at the point-of-care [22, 23]. Because RDT signal intensity is directly related to the number of biomarkers that reach the test line, one approach to improving the sensitivity of RDTs might be to add a larger blood sample volume (and thus more biomarkers) to the sample pad. If this approach enhanced RDT signal, adding a sample preparation step to concentrate the biomarkers would be of little value. The limits of this increased blood volume strategy was determined for the Paracheck *Pf* RDTs without modifying the volume of running buffer added to the test. RDT performance and lateral flow strip ‘clearing’ was evaluated over the 30-min time course recommended by the manufacturer. Clearing refers to the lateral flow strip returning to the original white background colour after a blood sample has flowed down the lateral flow strip and been absorbed into the wicking pad. If blood does not clear from the test, the lateral flow test generally does not develop properly, the test line often becomes masked by the blood, and antigen–antibody interactions at the test and control line may be inhibited. Blood sample volumes of 0, 5, 10, 15, 20, and 50 µL containing 200 parasites/µL were run on Paracheck Pf RDTs. After 30 min, the RDT with 5 µL blood completely cleared, with pixel intensity near the baseline value; the RDT with 10 µL blood partially cleared, with pixel intensity trending downward; and the RDTs with 15 µL or more blood did not clear, with pixel intensity trending upward or remaining high (Fig. 2a). In the representative images of the RDTs from the 30-min time point (Fig. 2b), it is demonstrated that the RDT performed with 5 µL blood developed properly, as no blood is visible on the sample pad or the area of the lateral flow strip around the test and control lines (left image). The RDT performed with the 10 µL sample is nearly cleared, as no blood is visible on the sample pad but has not cleared the test and control line area (second image from the left). In the RDTs performed with 15 and 20 µL, capillary flow was noticeably inhibited, as blood was left on the sample pad, the test lines were masked by the red colour of the blood, and the control lines did not develop properly (right two images). Time-lapse video of RDT clearance of each blood sample volume tested is available in the Additional file 2. These data demonstrate that delivering more HRPII to the lateral flow strip by simply increasing the sample volume applied to the test is not feasible using Paracheck Pf RDTs because of poor RDT clearance and performance when using blood volumes >10 µL.

### Effect of sample volume on bead-binding kinetics

**Fig. 3:**
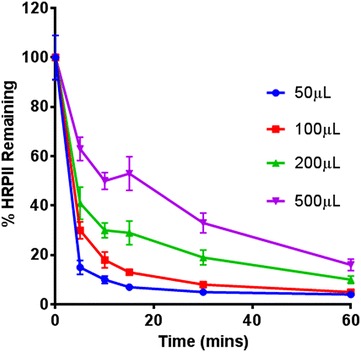
Percentage of total HRPII remaining in the lysed blood sample after mixing with Ni–NTA-functionalized magnetic beads over the course of 60 min from blood sample volumes of 25, 50, 100, and 250 µL containing 200 parasites/µL

RDT signal enhancement would be expected to increase as biomarkers are concentrated from larger sample volumes and delivered to the test line. However, during the magnetic bead capture step, if the number of magnetic beads remains constant, increasing the sample volume increases the diffusion distance that must be overcome for HRPII to bind the Ni–NTA on the surface of the magnetic beads, thus decreasing the probability of binding events for any given capture time. Therefore, it was predicted that for a fixed number of beads as sample volume increased, the time required to bind an equivalent portion of HRPII from the sample would also increase. To determine the effect of sample volume on biomarker binding kinetics, sample volumes of 25, 50, 100, and 250 µL containing 200 parasites/µL were mixed with 20 µL of beads for 5, 10, 15, 30, or 60 min, and the amount of HRPII remaining in the solution after the beads were removed was quantified by ELISA (Fig. 3). This volume of magnetic beads was selected based on previous work [19]. At 30 min, only 5 % of the initial HRPII remained in the 25-µL sample, whereas in the 250-µL sample, 30 % of the protein remained. Most of the protein was bound from the 25 and 50-µL samples after 1 h, where 10 and 16 % remained in the 100 and 250-µL samples, respectively. A fit of this collective sample volume and time data found the parameter *K* from Eq. 1 to be 21.53 μL/min (95 % confidence bounds: 17.49, 25.57; R^2^ = 0.9247). These data support the conclusion that increasing sample volume while fixing the number of beads increases the time required to bind a particular portion of available HRPII to the Ni–NTA-functionalized the bead surface.

### Effect of initial blood sample volume on limit of detection of Paracheck RDTs

To assess whether concentrating biomarkers from a larger sample volumes improves RDT performance, the limit of detection of this approach was determined by generating signal response curves over a range of parasite concentrations for each sample volume (see Additional file 1: Figure S2). Based on the analysis of these data, it was determined that the limit of detection improved when using magnetic beads to concentrate HRPII from larger sample volumes (Table 1). An enhancement factor, defined as the ratio of the limit of detection using the magnetically enhanced approach to the limit of detection of unenhanced Paracheck, was also calculated for each sample volume. Using a sample volume of 250 µL and a 30-min HRPII capture step, a 21-fold improvement over unenhanced Paracheck RDT performance was achieved, and more importantly, resulted in a limit of detection below 1 parasites/µL. At each sample volume tested, RDT signal enhancement was observed over an unenhanced RDT, yet less enhancement was observed at smaller sample volumes as fewer HRPII molecules were delivered to the lateral flow strip. Control RDTs were also run at each sample volume without parasites added to ensure that the method did not concentrate non-target proteins that could lead to false positives. For each control sample volume tested, the RDT reader did not read a value at the test line, as there was no signal at this region (see Additional file 1: Figure S3). While it is known that HRPII concentrations in vivo can be variable among different *P. falciparum* strains and is dependent on the developmental stage of the parasite, for these method development studies, dilution of stock D6 parasite culture provided a useful metric with minimal variability so that the focus of the studies were on device variability rather than sample-to-sample variability [24]. Additionally, a 200-fold dilution of 200 parasites/µL may not fully represent the complex physiological response of a malaria patient with a circulating infection of 1 parasite/µL, but representation of the enhancement method as such demonstrates how these RDTs can be utilized, as currently manufactured, to achieve PCR-like limits of detection.

**Table 1:** Limit of detection and enhancement factor of magnetic bead-enhanced RDTs relative to an unenhanced RDT

Sample volume (μL)	LOD (parsaites/μL)	Enhancement factor
5^a^	15.4	(Unenhanced)
25	7.9	1.9
50	5.5	2.8
100	1.7	9.1
250	0.73	21

^a^Control RDT performed according to manufacturer’s protocol

### Benefits and trade-offs of the magnetically enabled biomarker delivery approach

**Fig. 4:**
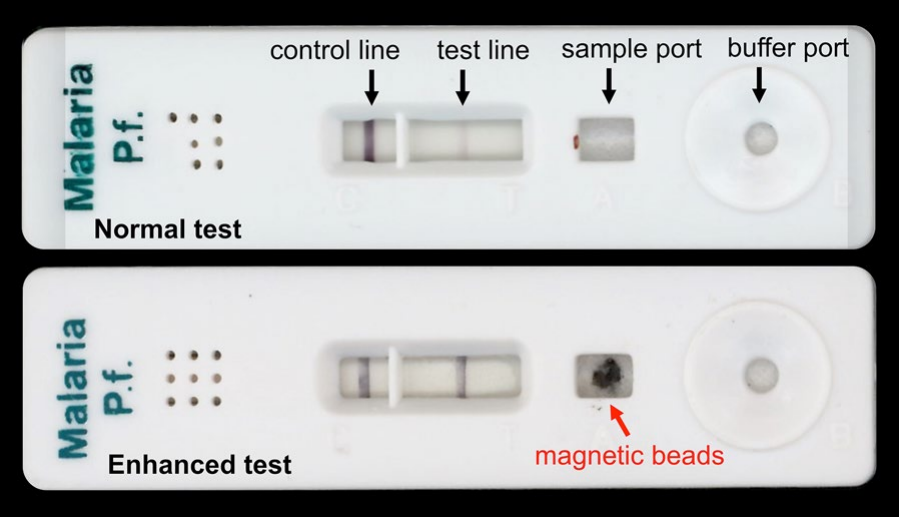
Paracheck RDTs before (*top*) and after (*bottom*) magnetic bead-based enhancement. The *top* RDT shows the result of applying a 5 µL blood sample containing 200 parasites/µL according to the manufacturer’s protocol. The *bottom* RDT shows enhancement at the test line after using the bead transfer device to apply HRPII-bound magnetic beads after mixing with a 100 µL sample containing 200 parasites/µL. HRPII is released from the deposited beads with imidazole-spiked running buffer

Because commercial RDTs are manufactured with buffer solutions, reagents and materials that are optimized for intracompatibility and test performance, it is remarkable that the magnetically enabled biomarker delivery approach does not impede RDT function. The compatibility of the approach with commercial RDTs is the result of a few interesting phenomena. The first is that the fluid dynamics and imidazole concentration of the running buffer are sufficient to release the HRPII from the blotted bead mound within the time frame required for the HRPII to bind to the gold nanoparticle conjugates as they flow up to the RDT test lines. The second is that despite being deposited directly on top of the lateral flow strip, as shown in Fig. 4, the Qiagen Agarose Ni–NTA magnetic beads do not appear to disrupt the flow of sample and buffer as the test develops. Thirdly, the addition of imidazole does not significantly impact the antibody binding, signal generation, or the general performance of the RDT (see Additional file 1: Figure S4). Also, it was found that for blood sample volumes in the range of 50–250 µL, the amount of blood component transferred to the test was <10 µL (see Additional file 1: Figure S5), which is within the allowable range of blood volumes determined in Fig. 2.

Despite the compatibility of this new approach with Paracheck Pf RDTs and the enhancement in RDT performance when using these HRPII bound magnetic beads, there are areas in which the method could be further improved. Given the expected concentration factors from the larger sample volumes, the maximum theoretical enhancement factors are greater than the experimentally determined values. For example, the 250-µL blood sample produced a 21-fold enhancement in Paracheck’s limit of detection, but based on the volume ratio (250–5 µL), the enhancement potential is 50-fold. One factor that contributes to this disparity is the trade-off between the volume of the blood sample and the time it takes to bind a certain percentage of the biomarkers present. The percentage of HRPII captured from the 250-µL blood sample after 30 min is 67 % (Fig. 3). For a fixed number of magnetic beads, the parameters under experimental control, namely time and volume, are inversely proportional (refer to Eq. 1), therefore, to increase the amount of HRPII captured on the bead surface, larger sample volumes will require longer incubation times. However, increasing the other factors contained in K (i.e., the number of beads, surface density of Ni–NTA, and the binding kinetics of HRPII to the bead surface) will also improve the capture efficiency for any given time and volume.

Effective transfer of biomarker-bound magnetic beads to the RDT is key to the performance of the enhancement method. The ‘blotting’ approach that is currently used to transfer bead from the dispensing tube to the lateral flow strip effectively and reproducibly transfers beads to the RDT. However, the amount of blood that is transferred to the RDT during blotting varies between 5 and 10 µL (10 and 20 µL of total lysed blood solution) depending on the user’s blotting technique (see Additional file 1: Figure S5). This variation in liquid transfer likely contributes to run-to-run signal error. Additionally, the efficiency of HRPII elution from the pellet of blotted beads may account for some of the losses. Elution efficiency may be improved by maximizing the interactions of the bead surface with imidazole by distributing the beads more evenly on the lateral flow strip or adding imidazole directly to the blotted beads. Future efforts will focus on minimizing the amount of residual sample transferred and maximizing bead surface interactions with imidazole.

Another important variable in some point-of-care settings is cost per assay. Commercial malaria RDTs cost US$0.55–$1.50 per test [2]. The additional cost of materials in the proposed method is dominated by the current low-volume cost of the Qiagen Ni–NTA magnetic beads (~US$2.20 per test). Based on the commercial availability of Ni–NTA bead options that are significantly less expensive than those used in this study, it is expect that the additional cost per assay can be reduced to much less than US$1.00. Despite this additional cost per assay, the limit of detection of the enhanced RDT method approaches that of current PCR-based strategies and, therefore, has the potential to significantly reduce the costs and infrastructure required for elimination campaigns.

## Conclusions

A simple magnetic bead-based sample preparation approach was shown to enhance a commercial malaria RDT by up to 21-fold, resulting in a limit of detection less than 1 parasite/µL without physically altering the RDT. The approach concentrates HRPII biomarkers from whole blood sample volumes that exceed the volume capacity of the RDT by capturing the HRPII from the samples using Ni–NTA functionalized magnetic beads, magnetically transferring the beads in a small volume to the RDT, and releasing the HRPII onto the RDT using imidazole-spiked running buffer. This approach features a simple, robust and low-cost format usable in settings with limited access to trained medical personnel and laboratory resources. Future work aims to streamline the sample collection tube integration with blood collection from a finger prick and modify the 3D-printed housing for ease-of-use with other RDT brands. It is anticipate that the improved performance of RDTs, due to delivery of more biomarkers from asymptomatic carriers, will result in an immediate impact in malaria elimination campaigns by enabling identification of asymptomatic patients.

## Additional files

10.1186/s12936-016-1448-6 Additional method details, images of the 3D printed transfer device, limit of detection curves at varying sample concentration, data on the effect of sample volume transfer and imidazole concentration on RDT performance, and a video of blood clearing from RDTs are presented in the additional files.
10.1186/s12936-016-1448-6 A time-lapse video illustrating how blood volume affects the clearance of rapid diagnostic tests. The video was recorded at a frame rate of 0.25 frames per second and a resolution of 1410 × 793; the displayed frame rate and resolution are 30 frames per second and 640 × 360, respectively. Image analysis was performed on each test after synchronizing the individual tests by the time that the buffer was added to a test’s buffer well.
