# Supplementary material for: Direct transfer of HRPII-magnetic bead complexes to malaria rapid diagnostic tests significantly improves test sensitivity
Source: Malar J. 2016 Aug 5;15:399. doi: 10.1186/s12936-016-1448-6 (PMC4975893; doi:10.1186/s12936-016-1448-6)
Supplement: Supplementary file 1 — 10.1186/s12936-016-1448-6 Additional method details, images of the 3D printed transfer device, limit of detection curves at varying sample concentration, data on the effect of sample volume transfer and imidazole concentration on RDT performance, and a video of blood clearing from RDTs are presented in the additional files. [file 12936_2016_1448_MOESM1_ESM.docx]

**Direct Transfer of *pf*HRPII-bound Magnetic Beads to Malaria Rapid Diagnostic Tests for Detection of One Parasite per Microliter of Blood**

Keersten M. Ricks,^1,†^ Nicholas M. Adams,^2,†^  Thomas F. Scherr,^2^ Frederick R. Haselton,^1,2^ David W. Wright,^1^*

^1^Department of Chemistry, Vanderbilt University, Nashville, TN 37235

^2^Department of Biomedical Engineering, Vanderbilt University, Nashville, TN 37235

†These authors contributed equally.

*To whom correspondence should be addressed: david.wright@vanderbilt.edu, +1-615-322-2636

**Keywords:** malaria, diagnostics, extraction, magnetic beads, biomarker concentration

**Supporting Information**

**Methods**

*Effect of Sample Volume on the Amount of Liquid Transferred to the RDT Sample Pad*

Lysed blood was prepared by mixing lysis buffer (100 mM sodium phosphate, 600 mM NaCl, 80 mM imidazole, 2% Triton X-100, pH 8) with blood volumes of 25, 50, 100, and 250 µL at a 1:1 ratio. Each sample volume was combined with 20 µL of Ni-NTA particles in a dispensing tube. Prior to blotting the particles on a Paracheck RDT, initial RDT weight was recorded and averaged from three measurements. The mBEADS device was then used to align the PCR tube with the sample pad above a magnet. After the magnetic beads were collected at the bottom of the tube for ~30 seconds, the tube was depressed ~2 mm until the open bottom of the dispensing tube contacted the surface of the sample deposition pad to dispense the beads. Immediately after blotting, the weight of the RDT was recorded. This process was repeated a total of three times for each of the four lysed blood sample volumes. The volume blotted was calculated as the difference in RDT weight after blotting versus before, divided by the density of lysed blood (1.072 g/mL).

*Effect of Imidazole on the Test Line Signal*

RDTs were run in triplicate using 5 µL of whole blood containing 200 parasite/µL and developed using Paracheck running buffer spiked to final imidazole concentrations of 50, 250, 500, 750, 1000 mM. A control test was performed using running buffer without imidazole added (as supplied by the manufacturer). Each RDT was developed for 30 minutes prior to reading the signal at the test line using the Qiagen ESEQuant LFR.


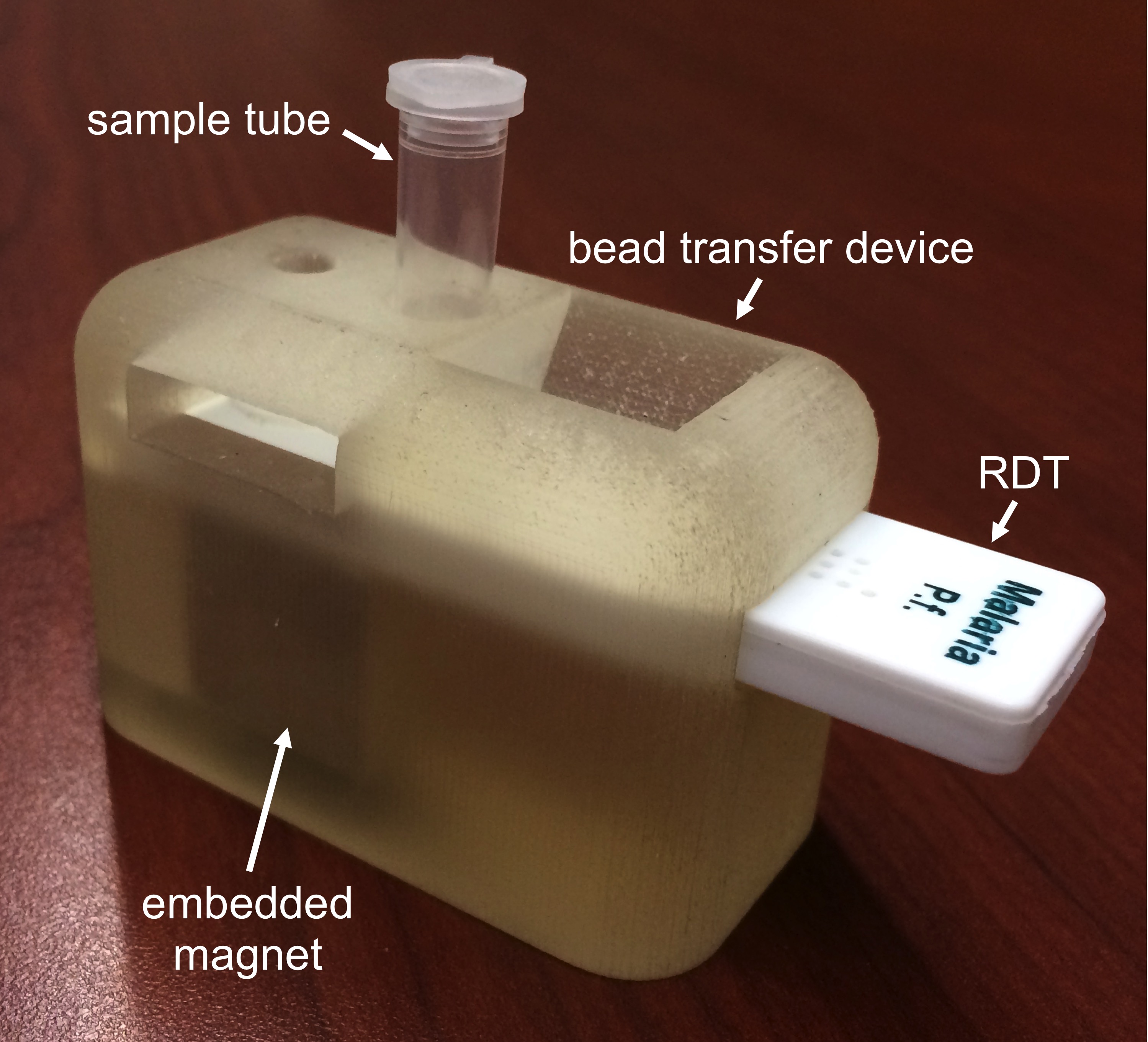


Supporting Information Figure 1. 3D printed magnetic bead transfer device. The device was designed to integrate with the Paracheck RDT form factor and promote ease of magnetic bead transfer by aligning a fixed magnet (within the device) with the sample pad of the lateral flow strip.


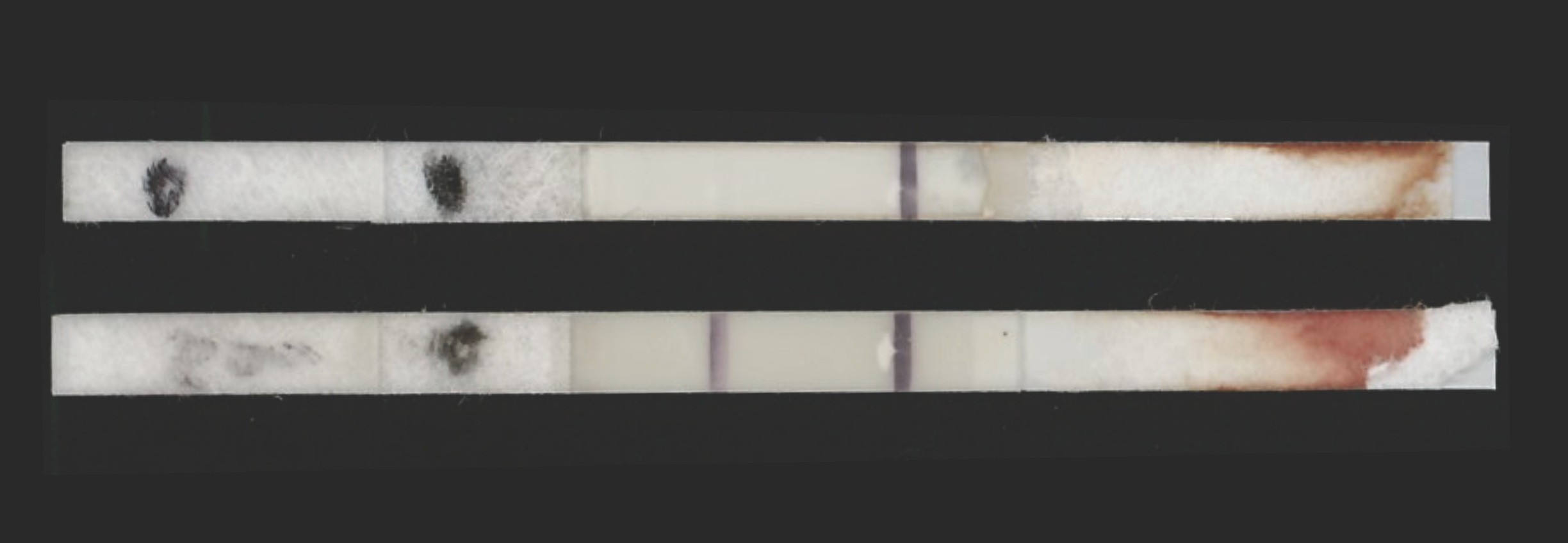

Supporting Information Figure 2. Effect of blood sample volume on the limit of detection of the Paracheck malaria RDT, after biomarker transfer using Ni(II)NTA magnetic beads. Using the Qiagen ESEQuant, peak area at the test line (indicative of the presence of HRPII) was plotted as a function of the concentration of parasites in the blood sample.

Supporting Information Figure 3. Representative image of a negative blood sample (top lateral flow strip) and a positive blood sample at 200 parasites/µL (bottom lateral flow strip) after biomarker transfer from a 100µL sample using Ni(II)NTA magnetic beads. After bead transfer and biomarker release with imidazole spiked running buffer, the lateral flow strips seen above were taken out of the plastic RDT housing. No signal was detected at the test line from the negative RDT when scanned using the Qiagen ESE Quant RDT reader, indicating the enhancement method does not induce false positives. These results with negative samples were observed across all sample volumes tested.

Supporting Information Figure 4. Effect of imidazole on the test line of Paracheck RDTs. All RDTs were processed at 200 parasites/µL. No significant changes in the test line signal as a function of increasing imidazole in the running buffer were observed.

Supporting Information Figure 5. Effect of sample volume on the volume of lysed blood blotted on the sample pad of a Paracheck RDT. Note that because the lysed blood sample is a 1:1 ratio of lysis solution to blood, the amount of blood component transfer is half of the measured volume blotted on the pad (mean ± SD, n = 3). Values are not significantly different based on an unpaired t-test (p>0.05).
